# Supplementary material for: Innate Immune Pathways Promote Oligodendrocyte Progenitor Cell Recruitment to the Injury Site in Adult Zebrafish Brain
Source: Cells. 2022 Feb 2;11(3):520. doi: 10.3390/cells11030520 (PMC8834209; doi:10.3390/cells11030520)
Supplement: Supplementary file 1 [file cells-11-00520-s001.zip › Sanchez-Gonzalez_et_al_Supplementary_Material/Sanchez-Gonzalez_et_al_Suppl_Figures.pdf]

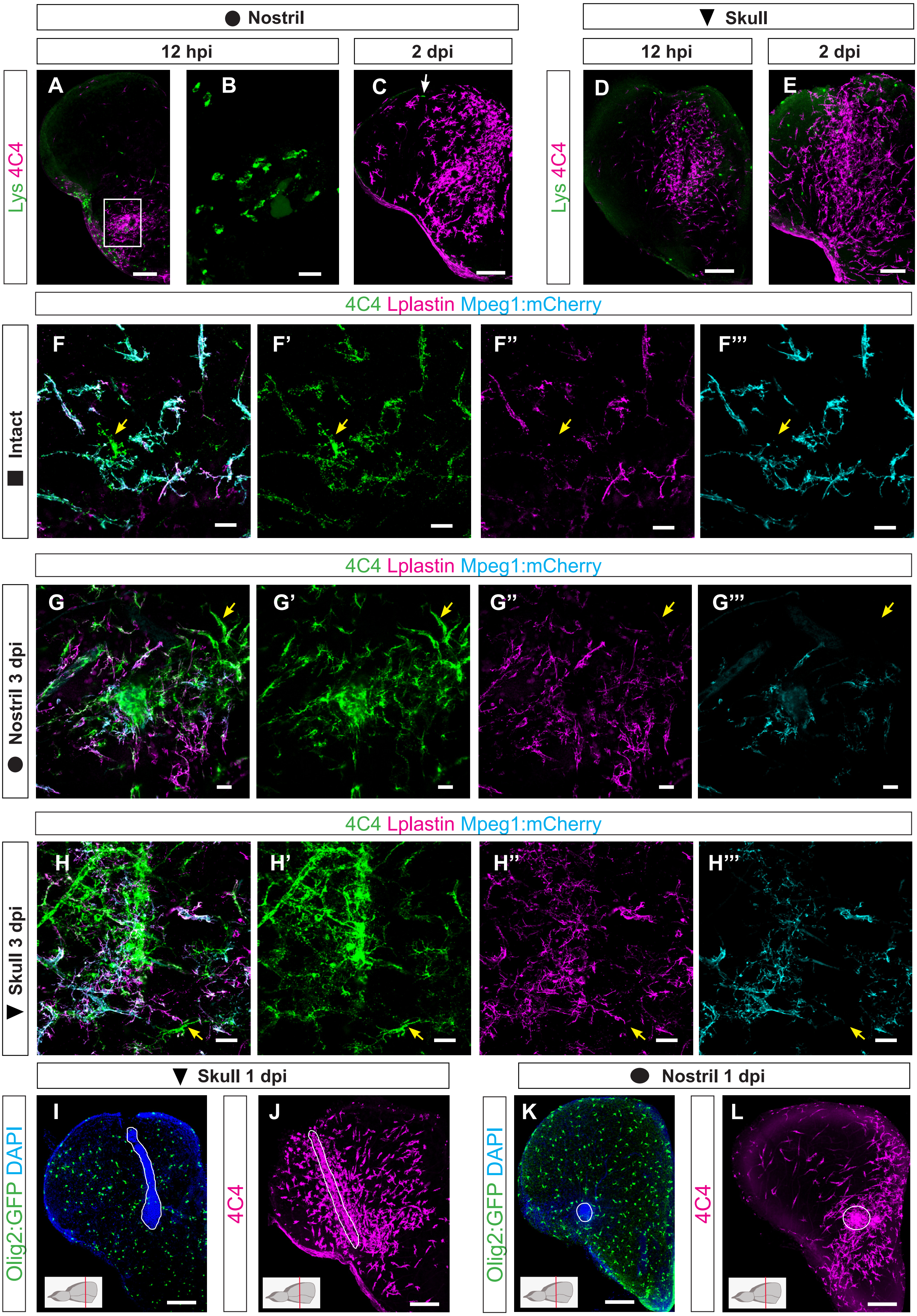

Supplementary Figure S1

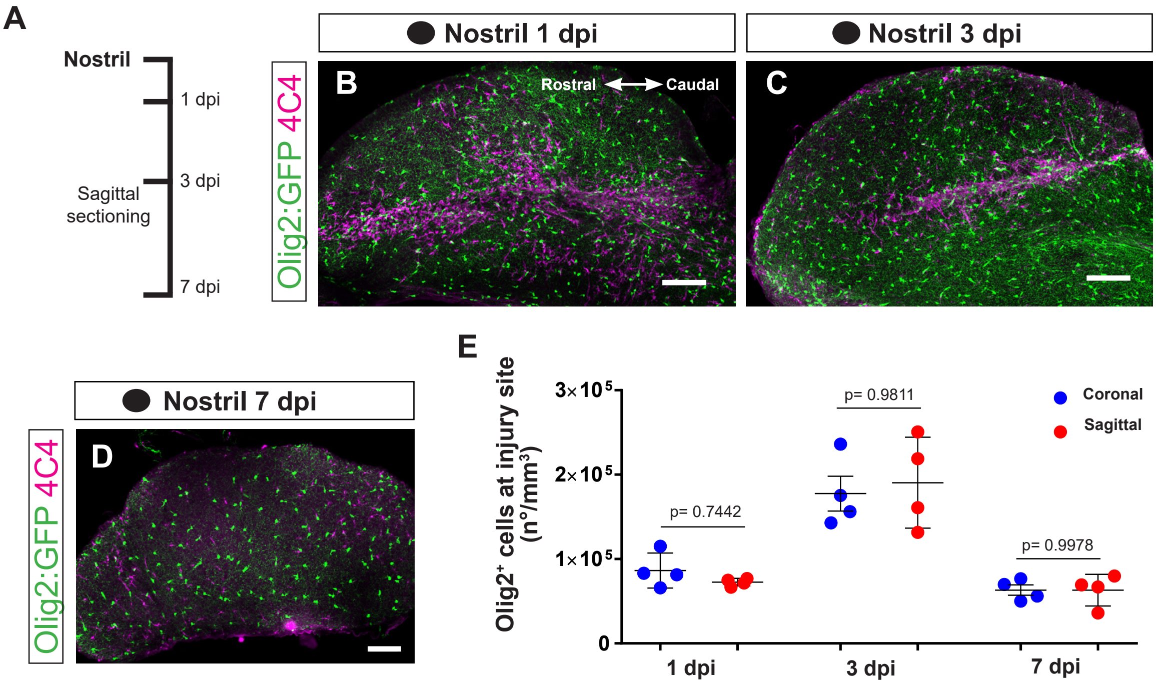

Sanchez-Gonzalez et al., 2021 Supplementary Figure S2

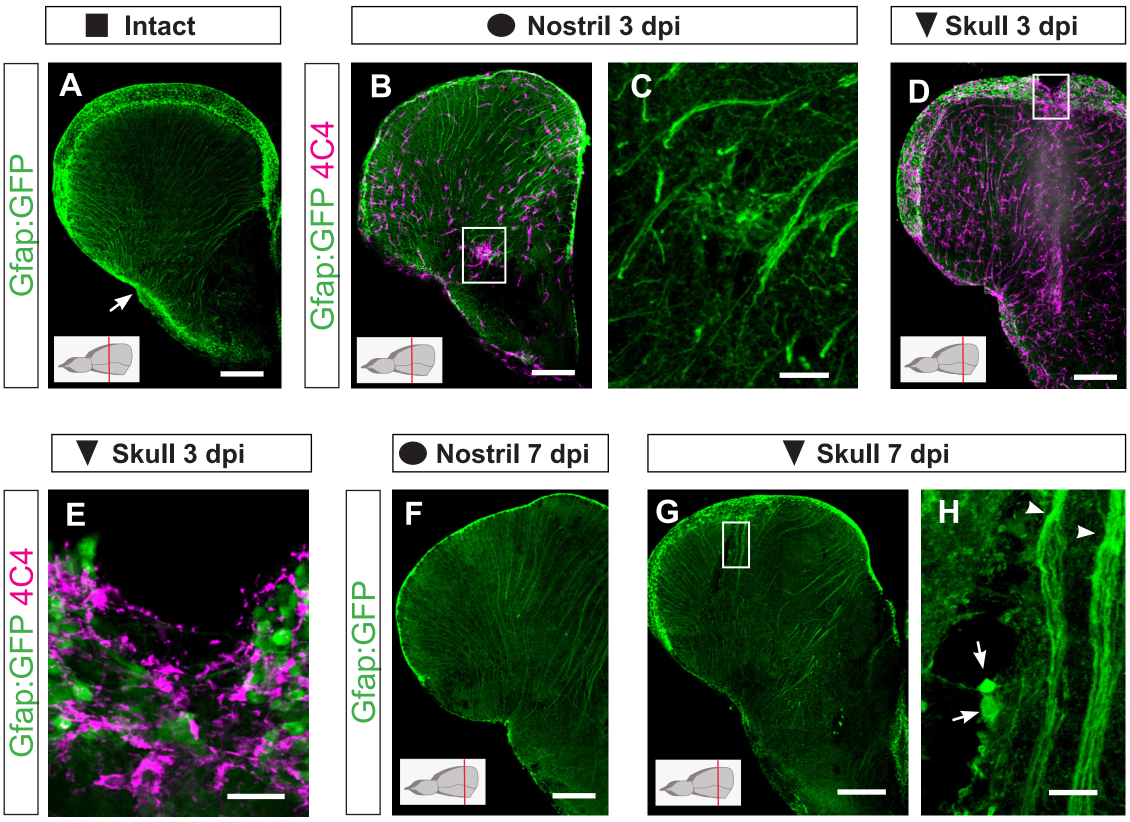

Sanchez-Gonzalez et al., 2021 Supplementary Figure S3

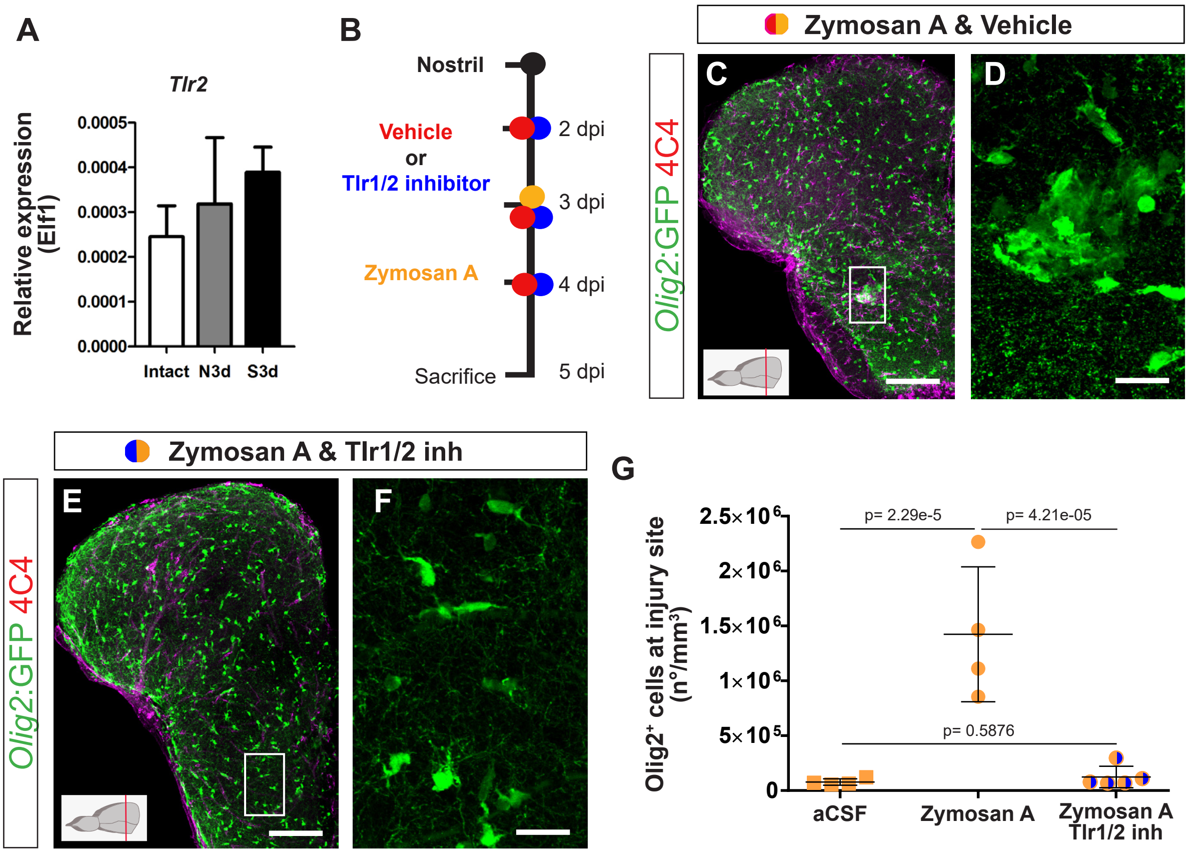

Sanchez-Gonzalez et al., 2021 Supplementary Figure S4

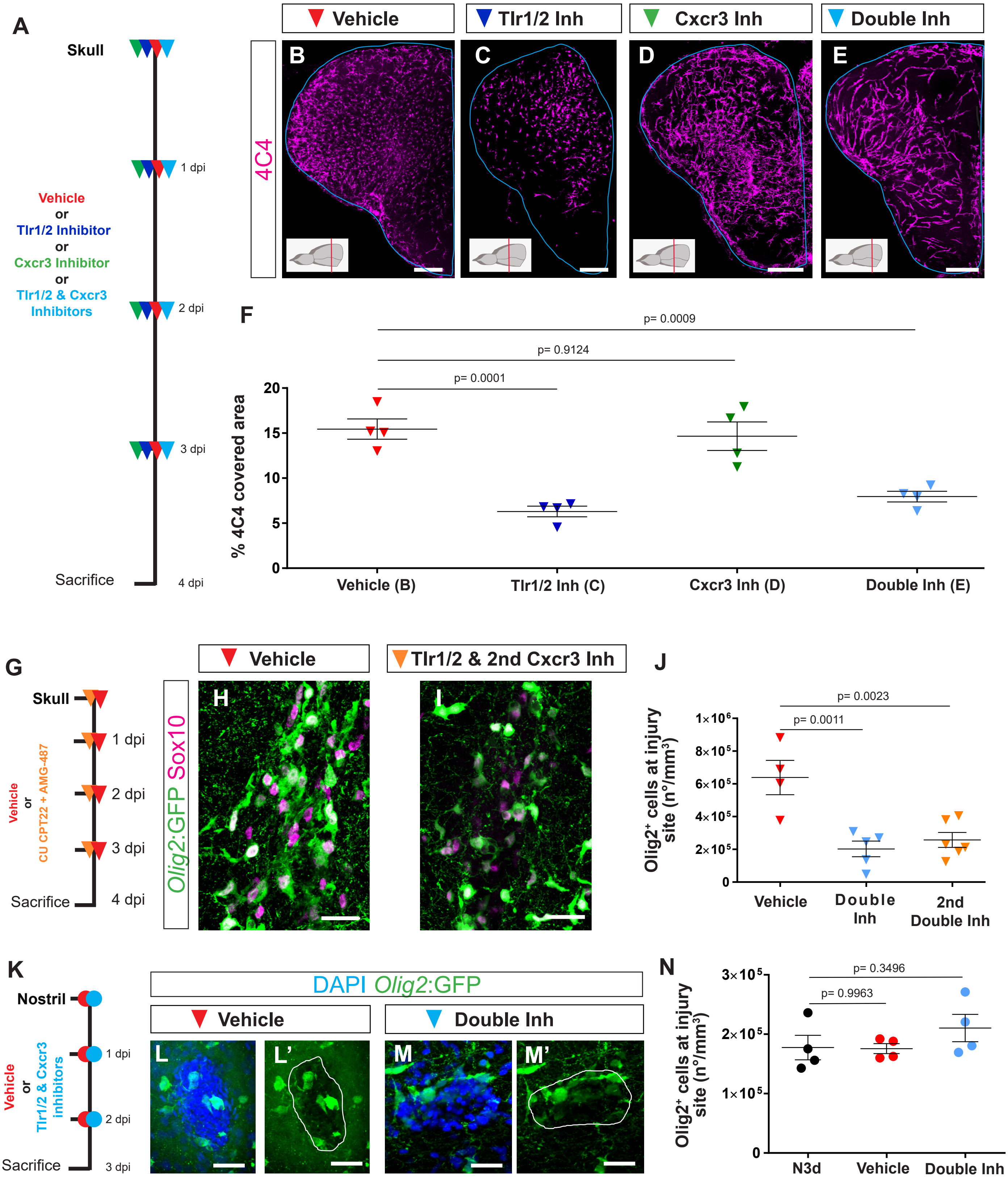

Sanchez-Gonzalez et al., 2021 Supplementary Figure S5

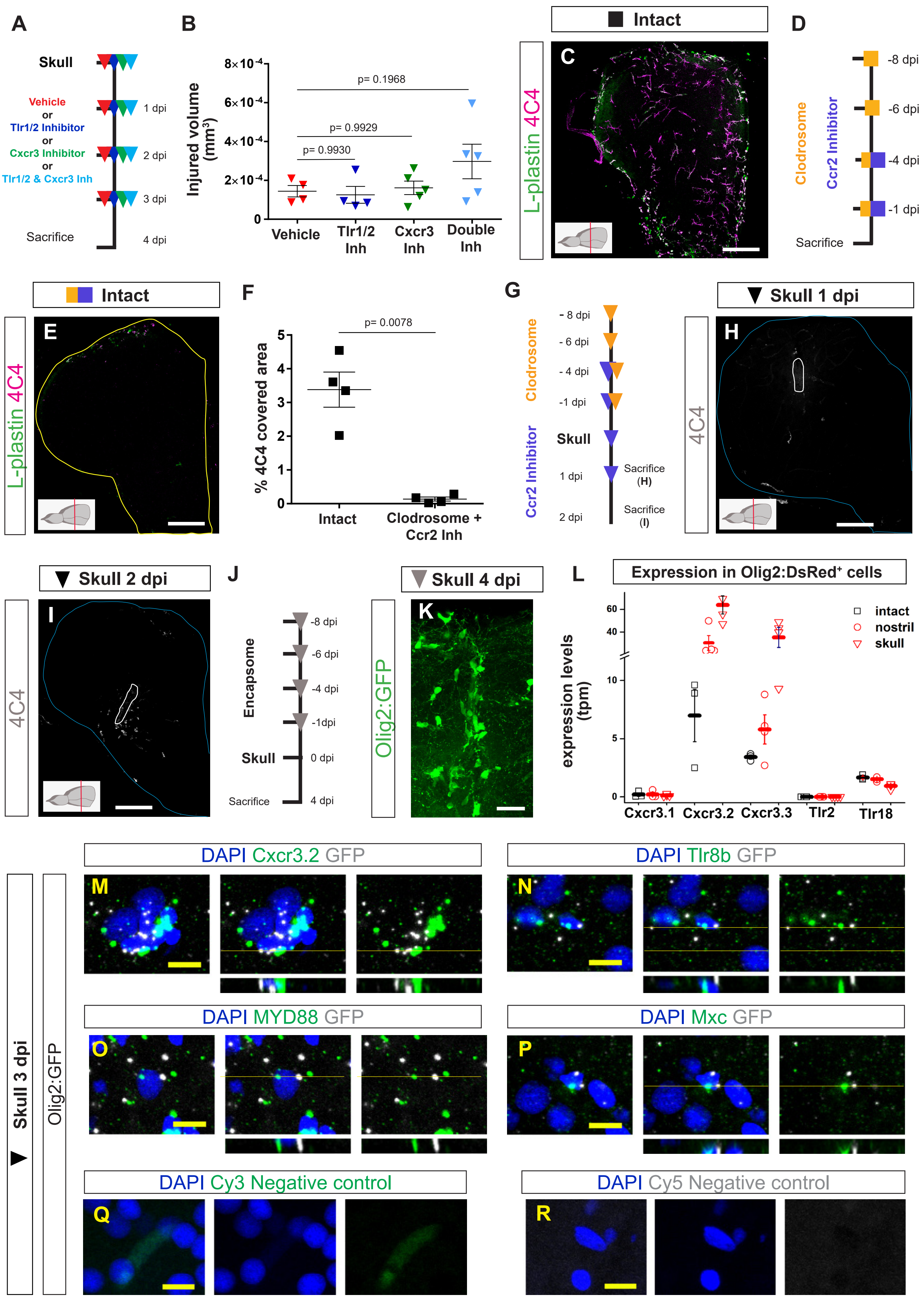

Sanchez-Gonzalez et al., 2021 Supplementary Figure S6

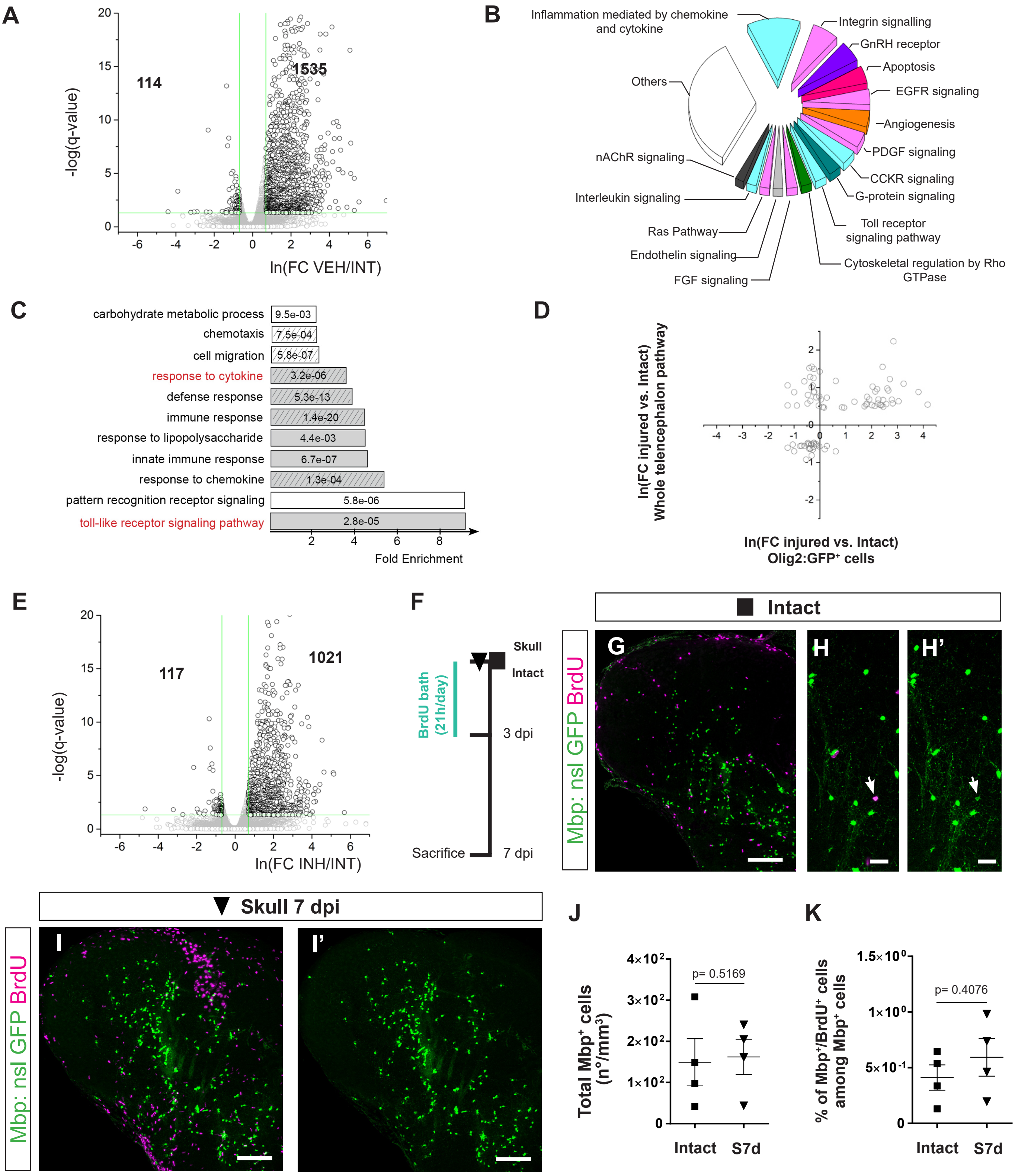

Sanchez-Gonzalez et al., 2021 Supplementary Figure S7

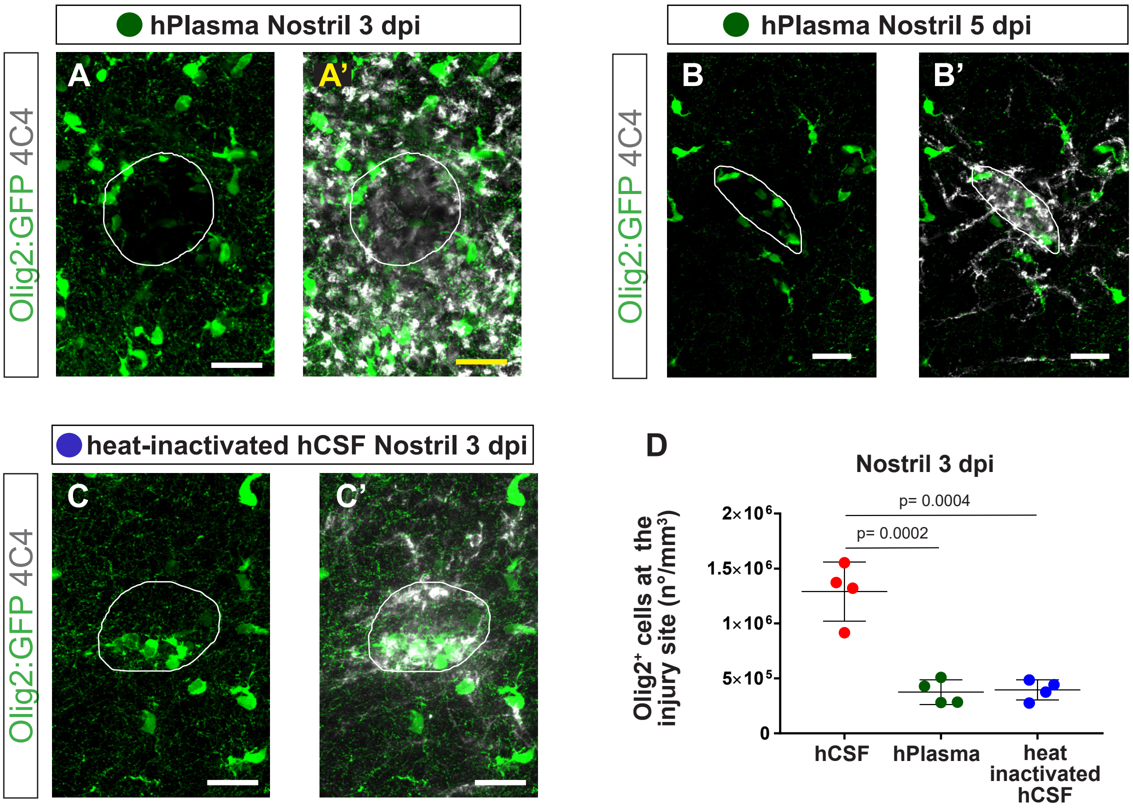

Sanchez-Gonzalez et al., 2021 Supplementary Figure S8

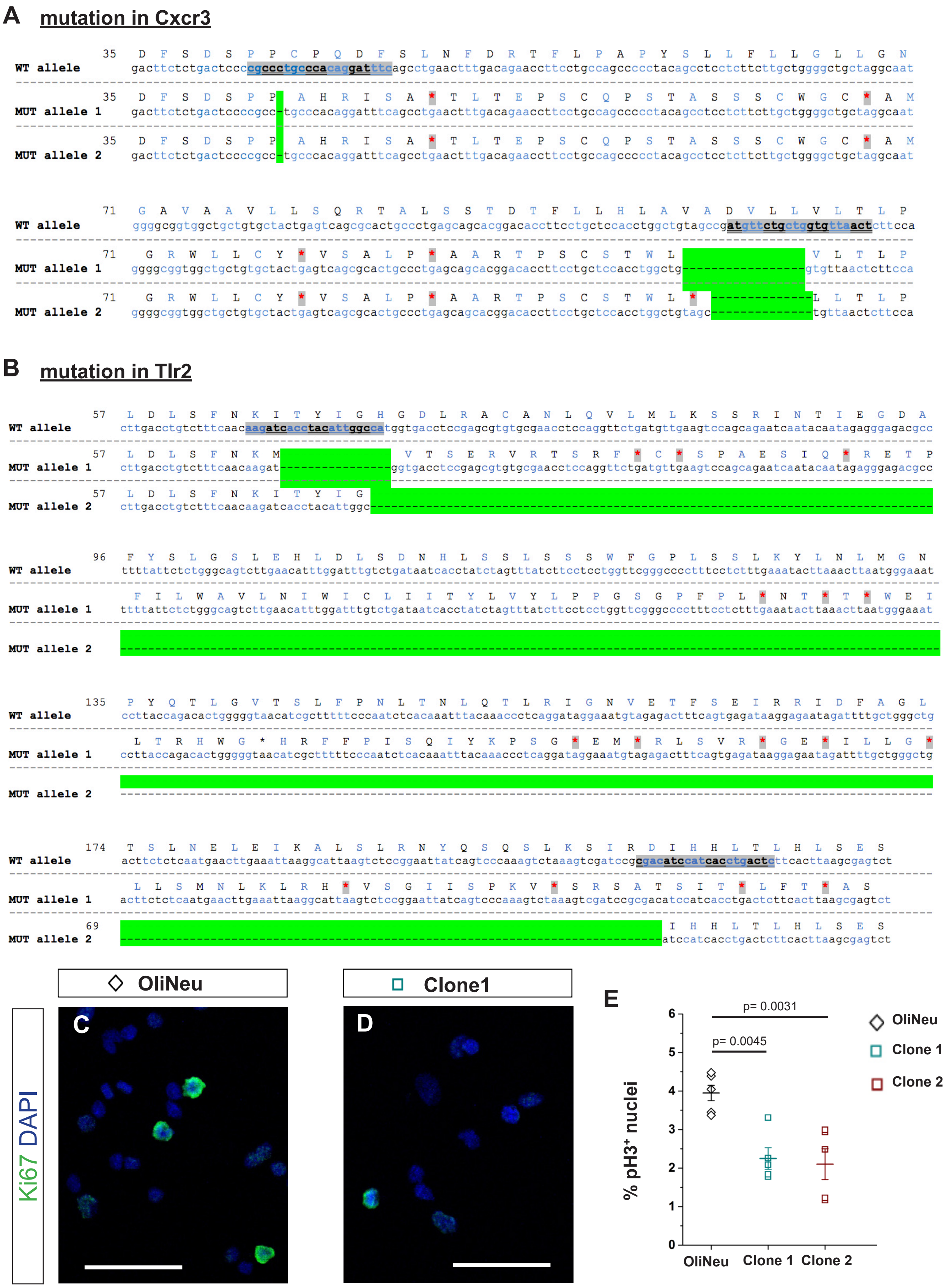

Sanchez-Gonzalez et al., 2021 Supplementary Figure S9
