# Supplementary material for: Innate Immune Pathways Promote Oligodendrocyte Progenitor Cell Recruitment to the Injury Site in Adult Zebrafish Brain
Source: Cells. 2022 Feb 2;11(3):520. doi: 10.3390/cells11030520 (PMC8834209; doi:10.3390/cells11030520)
Supplement: Supplementary file 1 [file cells-11-00520-s001.zip › Sanchez-Gonzalez_et_al_Supplementary_Material/Sanchez-Gonzalez_et_al_Suppl_Tables.pdf]

Table S1. List of all the primary antibodies used in this study, and the correspondent information about each antibody.

Antigen	Source	Dilution	Host, isotype	Pre-treatment
anti-fish leukocytes (4C4)	Health Protection Agency Culture Collections 92092321, 7.4.C4	1:500	Mouse IgG1	-
BrdU	Serotec (MCA2060)	1:200	Rat, IgG2a	2N HCl 30min
GFP	Aves lab (GFP-1020)	1:700	Chicken, IgY	-
Sox10	Genetex (GTX128374)	1:2000	Rabbit	-
HuC/HuD	Abcam (ab210554)	1:500	Rabbit	
L-plastin	Genetex (GTX114524)	1:200	Rabbit	Dako target retrieval solution (S1699)
phospho-Histone H3	Millipore (Cat# 06-570)	1:500	Rabbit	
Ki67	Thermofisher (Cat# 14-5698-80)	1:500	Rat	

Table S2. List of primers used in this study for RT-qPCR.

**Target**	**Forward (5'→3')**	**Reverse (3'→5')**
Tlr2	ACCTGCTCCAATCTTCAGCTC	TGCTTTCAAGCTCCCGTTCT

Table S3. List of recombinant proteins used for the screening in Figure 7G (www.rndsystems.com).

Human recombinant protein	Concentrations	Catalog number
TGFbeta	0.04 ng/mL; 0.12 ng/mL; 0.2 ng/mL	628-LK-025
Ccl17	2 ng/mL; 4 ng/mL; 6 ng/mL	364-DN-025
SDF	4 ng/mL; 16 ng/mL; 24 ng/mL	6448-SD-025
SCF	1 ng/mL; 2.5 ng/mL; 5 ng/mL	255-SC-010
Ccl5	1 ng/mL; 2.5 ng/mL; 5 ng/mL	278-RN-010
PDGFbeta	1.5 ng/mL; 3.5 ng/mL; 6 ng/mL	AFL220-010
MOSM	0.05 ng/mL; 0.17 ng/mL; 0.3 ng/mL.	295-OM-010
Ccl15	0.2 ng/mL; 4 ng/mL; 8 ng/mL.	628-LK-025
Cxcl9	0.1 µg/mL; 0.2 µg/mL; 0.4 µg/mL	392-MG-010
MCSF	0.5 ng/mL; 1 ng/mL; 1.5 ng/mL	216-MC-005
Ccl7	0.02 µg/mL; 0.1 µg/mL; 0.5 µg/mL	282-P3-010
Ccl8	0.03 µg/mL; 0.05 µg/mL; 0.1 µg/mL	281-CP-010
INF gamma	0.15 ng/mL; 0.5 ng/mL; 0.75 ng/mL	285-IF-100
IL-15	0.3 ng/mL; 1.3 ng/mL; 2.6 ng/mL	247-ILB-005
IL-12	0.01 ng/mL; 0.02 ng/mL; 0.05 ng/mL	219-IL-005
IL-10	0.15 ng/mL; 0.5 ng/mL; 0.75 ng/mL	217-IL-005
IL-8beta	0.5 ng/mL; 1.25 ng/mL; 2.5 ng/mL	208-IL-010
IL-3	0.02 ng/mL; 0.05 ng/mL; 0.1 ng/mL	203-IL-010
IL1-beta	1 pg/mL; 6 pg/mL; 12 pg/mL	201-LB-005
IGF-1	0.3 ng/mL; 1 ng/mL; 1.5 ng/mL	AFL291-200
EGF	20 pg/mL; 60 pg/mL; 100 pg/mL	AFL236-200
Cxcl11	1 ng/mL; 2.5 ng/mL; 5 ng/mL	672-IT-025
Cxcl10	0.03 ug/mL; 0.1 ug/mL; 0.18 ul/mL	266-IP-010
Cxcl5	3 ng/mL; 9 ng/mL; 15 ng/mL	254-XB-025
Cxcl1	1 ng/mL; 0,15 µg/mL; 0.3 µg/mL	275-GR-010
Ccl22	0.5 ng/mL; 1.5 ng/mL; 3 ng/mL	336-MD-025
Ccl2	5 ng/mL; 25 ng/mL.; 30 ng/mL.	279-MC-010
Ccl1	1.5 ng/mL; 4.5 ng/mL; 9 ng/mL	272-I-010
Angiogenin	2.5 ug/mL; 5 ug/mL; 10 ug/mL	265-AN-250

Table S4. Microarray data illustrating the GO Terms Biological Processes enriched in genes commonly or exclusively regulated in skull and nostril injury at different time points (related to Figure 2B).

Commonly regulated GO terms 1dpi (Figure 2B)

GO Term	Number of genes	p-value	Genes	Fold Enrichment
GO:0051549~ positive regulation of keratinocyte migration	3	0.002554	MMP9, SERPINE1, HBEGF	38.19
GO:0051085~ chaperone mediated protein folding requiring cofactor	3	0.006889	ERO1L, H2-DMA, CD74	23.50
GO:0060397~ JAK-STAT cascade involved in growth hormone signaling pathway	3	0.006889	PTPN6, SOCS3, STAT3	23.50
GO:0019886~ antigen processing and presentation of exogenous peptide antigen via MHC class II	3	0.007986	IFI30, H2-DMA, CD74	21.82
GO:0051279~ regulation of release of sequestered calcium ion into cytosol	3	0.007986	CYBA, PTPN6, CORO1A	21.82
GO:0016064~ immunoglobulin mediated immune response	4	0.001404	C4B, INPP5D, H2-DMA, CD74	17.71

GO:0030574~ collagen catabolic process	4	0.001593	CTSK, MMP9, CTSS, MMP14	16.97
GO:0007159~ leukocyte cell-cell adhesion	4	0.001797	ITGAL, ITGB2L, FERMT3, ITGB1	16.29
GO:0035987~ endodermal cell differentiation	4	0.002505	ITGB2L, MMP9, MMP14, FN1	14.54
GO:0051603~ proteolysis involved in cellular protein catabolic process	8	1.48E-06	SCPEP1, CTSK, PSMB7, LGMN, CTSA, CTSC, CTSS, CTSH	14.04
GO:0070527~ platelet aggregation	5	4.51E-04	PTPN6, FERMT3, CSRP1, CLIC1, MYH9	13.76
GO:0042517~ positive regulation of tyrosine phosphorylation of Stat3 protein	4	0.003061	HES5, IL6ST, STAT3, CSF1R	13.57
GO:0048147~ negative regulation of fibroblast proliferation	4	0.003686	MMP9, IFI30, C1QL4, FTH1	12.73
GO:0007017~ microtubule-based process	4	0.007431	TUBA8, TUBA3B, TUBB6, TUBB4B	9.93
microtubule-based process	6	4.31E-04	TNFRSF1A, GPSM3, SERPINE1, TGM2, CTSS, ADAM8	9.40
GO:0007160~ cell-matrix adhesion	6	0.001055	ITGAL, ITGB2L, ITGB7, ADAM8, ITGB1, FN1	7.73
GO:0071260~ cellular response to mechanical stimulus	6	0.001247	CYBA, TNFRSF1A, IRF1, CNN2, FAS, ITGB1	7.45

GO:0071347~ cellular response to interleukin-1	6	0.001543	SERPINE1, IRF1, PYCARD, FAS, KLF2, FN1	7.10
GO:0050728~ negative regulation of inflammatory response	6	0.001709	TNFAIP6, TNFRSF1A, SOCS3, NLRP3, ADA, LGALS9	6.94
GO:0007229~ integrin-mediated signaling pathway	6	0.00218	ITGAL, ITGB2L, FERMT3, ITGB7, ADAM8, ITGB1	6.57
GO:0048661~ positive regulation of smooth muscle cell proliferation	5	0.00781	CYBA, HES5, HMOX1, TGM2, HBEGF	6.36
GO:0034097~ response to cytokine	5	0.009247	STAT6, CORO1A, IL6ST, TIMP2, STAT3	6.06
GO:0030335~ positive regulation of cell migration	12	6.16E-06	TNFAIP6, CORO1A, CCR1, FERMT3, HBEGF, MMP14, MCAM, ITGB1, CTSH, CSF1R, F2R, FN1	5.90
GO:0030036~ actin cytoskeleton organization	8	5.39E-04	CORO1A, PFN3, RAC2, WASF2, FHL3, WIPF1, CSRP1, RHOG	5.65
GO:0019221~ cytokine-mediated signaling pathway	8	5.85E-04	STAT6, TNFRSF1A, PTPN6, IL6ST, SOCS3, CSF2RB, STAT3, CSF1R	5.58
GO:0045766~ positive regulation of angiogenesis	6	0.00694	TNFRSF1A, CYBB, MMP9, HMOX1, SERPINE1, CTSH	5.00
GO:0032355~ response to estradiol	6	0.008455	TXNIP, SOCS3, GRN, ANXA1, DNMT1, STAT3	4.77

GO:0006954~ inflammatory response	16	1.78E-06	C5AR2, C4B, CCR1, ANXA1, NLRP3, TNFRSF1A, CYBA, CYBB, ITGB2L, LTB4R2, PYCARD, PARP4, FAS, ADAM8, CSF1R, F2R	4.66
GO:0001525~ angiogenesis	10	6.18E-04	HMOX1, WASF2, SERPINE1, HBEGF, MYH9, ADAM8, MMP14, MCAM, FN1, ANXA2	4.22
GO:0006955~ immune response	13	7.48E-05	FYB, C7, TNFRSF1A, ENPP1, CCR1, IRF8, FAS, CTSS, H2-DMA, NFIL3, FTH1, CD74, LCP2	4.13
GO:0042127~ regulation of cell proliferation	9	0.001779	TXNIP, STAT6, TNFRSF1A, GM4907, SERPINE1, ANXA1, DNMT1, CNN2, FAS	4.03
GO:0002376~ immune system process	15	2.34E-05	MBL2, ANXA1, IFI30, NLRP3, C1QC, LGALS9, CD74, MARCO, CFP, C1QB, PYCARD, IRF1, INPP5D, H2-DMA, CSF1R	4.00
GO:0007568~ aging	7	0.009475	SOCS3, DNMT1, CTSC, FAS, TIMP2, ADA, STAT3	3.87
GO:0042493~ response to drug	13	2.82E-04	TXNIP, CYBA, CYBB, SOCS3, MMP9, ANXA1, NPPC, DNMT1, FAS, TIMP2, ITGB1, ADA, STAT3	3.58
GO:0007155~ cell adhesion	16	8.57E-05	ITGAL, OLFM4, FERMT3, MCAM, MYH9, ITGB1, ADA, TNFAIP6, ITGB2L, HES5, FAT4, ITGB7, CD22, MFAP4, THBS2, FN1	3.35
GO:0045087~ innate immune response	13	6.06E-04	MARCO, CFP, MBL2, CYBA, C1QB, CYBB, C4B, ANXA1, IRF1, PYCARD, NLRP3, C1QC, CSF1R	3.29
GO:0010628~ positive regulation of gene expression	11	0.006141	TNFRSF1A, ATF3, ACTA2, SERPINE1, DNMT1, CNN2, NFIL3, CTSH, LGALS9, STAT3, FN1	2.79

GO:0006508~ proteolysis	15	0.001648	SCPEP1, LGMN, MMP9, CTSA, TRY4, CTSS, NAPSA, MMP14, CTSK, PSMB7, ADAMTS8, HTRA1, CTSC, ADAM8, CTSH	2.64
GO:0055114~ oxidation-reduction process	15	0.006708	ME1, STEAP4, GLUD1, IFI30, MSRB2, FTH1, RDH12, CYBA, DHRS3, CYBB, TXNDC2, HMOX1, CYP2B19, ERO1L, GLRX	2.25

Commonly regulated GO terms 2dpi (Figure 2B)

**GO Term**	**Number of genes**	**p-value**	**Genes**	**Fold Enrichment**
GO:0000122~ negative regulation of transcription from RNA polymerase II promoter	16	0.006556	TXNIP, HCLS1, PPARG, SPI1, JUNB, STAT6, HHEX, ATF3, CRY2, HES5, HSF1, JUN, IRF8, DNMT1, CRY1, SCRT1	2.18
GO:0045944~ positive regulation of transcription from RNA polymerase II promoter	22	9.05E-04	KLF9, EGR4, MAFB, HCLS1, PPARG, SPI1, IGF2, NPAS4, NLRP3, JUNB, STAT6, FOS, HHEX, MEIS2, ATF3, HSF1, HES5, JUN, IRF1, TNIP2, FOXD1, TOP2A	2.21
GO:0007155~ cell adhesion	14	0.001086	ITGAE, FERMT3, MCAM, ADA, IGSF11, LGALS3BP, ITGB2L, HES5, FAT4, PECAM1, CD22, CSF3R, MFAP4, FN1	2.91
GO:0042493~ response to drug	11	0.003742	TXNIP, TYMS, FOS, LYN, MMP9, JUN, NCKAP1L, DNMT1, IGF2, JUNB, ADA	3.00

GO:0006508~ proteolysis	19	1.75E-05	SCPEP1, USP8, CNDP2, MMP9, CTSA, GZMB, TRY4, CTSS, NAPSA, MMP14, PSMB7, CTSK, PSMA6, TPP1, CTSC, LTA4H, ADAM8, CTSH, DPP7	3.31
GO:0045087~ innate immune response	14	1.86E-04	LYN, TLR13, NLRP3, C1QC, TLR8, MARCO, C1QA, CFP, C1QB, PYCARD, IRF1, FCER1G, MR1, CSF1R	3.51
GO:0032496~ response to lipopolysaccharide	8	0.004674	FOS, NLRC3, HSF1, JUN, DNMT1, JUNB, LGALS9, F2R	3.87
GO:0006955~ immune response	13	8.34E-05	FYB, C7, ENPP1, CCR1, IRF8, H2-AB1, TNFSF12, CTSS, MR1, H2-DMA, FTH1, H2-DMB2, LCP2	4.09
GO:0006954~ inflammatory response	15	9.98E-06	PIK3CG, C5AR2, LYN, CCR1, PPARG, TLR13, NLRP3, TLR8, ITGB2L, LTB4R2, PYCARD, TNIP2, ADAM8, CSF1R, F2R	4.33
GO:0030335~ positive regulation of cell migration	10	2.21E-04	CORO1A, LYN, CCR1, FERMT3, MMP14, MCAM, CTSH, CSF1R, F2R, FN1	4.87
GO:0001525~ angiogenesis	12	2.86E-05	PIK3CG, JUN, HMOX1, PECAM1, TNFSF12, ADAM8, MMP14, MCAM, ARHGAP24, GJA5, FN1, ANXA2	5.01
GO:0002376~ immune system process	19	4.50E-08	PIK3CG, LYN, TLR13, IFI30, H2-AB1, NLRP3, C1QC, LGALS9, TLR8, MARCO, CFP, C1QA, C1QB, PYCARD, IRF1, MR1, INPP5D, H2-DMA, CSF1R	5.02

GO:0006979~ response to oxidative stress	7	0.001999	TXNIP, GPX2, MMP9, HMOX1, CYGB, GPX8, MMP14	5.34
GO:0007623~ circadian rhythm	6	0.005282	TYMS, CRY2, CLDN4, KLF9, JUN, CRY1	5.35
GO:0006935~ chemotaxis	7	0.001127	PIK3CG, C5AR2, LYN, RAC2, CCR1, NCKAP1L, LGALS9	5.97
GO:0007229~ integrin-mediated signaling pathway	6	0.002289	ITGB2L, PLEK, FERMT3, ITGAE, FCER1G, ADAM8	6.50
GO:0034097~ response to cytokine	6	0.00146	STAT6, TYMS, FOS, CORO1A, JUN, JUNB	7.19
GO:0009612~ response to mechanical stimulus	6	5.23E-04	TXNIP, MEIS2, JUN, PPARG, MMP14, JUNB	9.02
GO:0019882~ antigen processing and presentation	5	0.001979	H2-AB1, CTSS, MR1, H2-DMA, H2-DMB2	9.33
GO:0009314~ response to radiation	4	0.004913	IL1R1, JUN, IGF2, JUNB	11.51
GO:0032570~ response to progesterone	5	6.36E-04	TXNIP, TYMS, FOS, CLDN4, JUNB	12.59
GO:0007249~ I-kappaB kinase/NF-kappaB signaling	4	0.003804	NLRC3, IRF1, TNIP2, TLR8	12.59
GO:0048147~ negative regulation of fibroblast proliferation	4	0.003804	MMP9, IFI30, C1QL4, FTH1	12.59
GO:0070527~ platelet aggregation	5	4.71E-04	PTPN6, PLEK, FERMT3, CSRP1, CLIC1	13.61

GO:0051603~ proteolysis involved in cellular protein catabolic process	8	1.60E-06	SCPEP1, CTSK, PSMB7, PSMA6, CTSA, CTSC, CTSS, CTSH	13.89
GO:0035987~ endodermal cell differentiation	4	0.002586	ITGB2L, MMP9, MMP14, FN1	14.39
GO:0045453~ bone resorption	4	0.001856	CTSK, RAC2, TPP1, CTSS	16.11
GO:0030574~ collagen catabolic process	4	0.001645	CTSK, MMP9, CTSS, MMP14	16.79
GO:0030833~ regulation of actin filament polymerization	4	0.001645	ARPC1B, CORO1A, PFN3, HCLS1	16.79
GO:0051279~ regulation of release of sequestered calcium ion into cytosol	3	0.008161	PTPN6, CORO1A, LYN	21.58

GO:0001913~ T cell mediated cytotoxicity	3	0.007041	GZMB, CTSC, CTSH	23.24
GO:0045576~ mast cell activation	3	0.007041	FYB, FCER1G, LCP2	23.24
GO:0097067~ cellular response to thyroid hormone stimulus	3	0.007041	KLF9, CTSS, CTSH	23.24
GO:0019886~ antigen processing and presentation of exogenous peptide antigen via MHC class II	5	8.52E-06	IFI30, FCER1G, H2-AB1, H2-DMA, H2-DMB2	35.97
GO:0032611~ interleukin-1 beta production	3	0.001971	PYCARD, MR1, NLRP3	43.16

Commonly regulated GO terms 3dpi (Figure 2B)

GO Term	Number of genes	p-value	Genes	Fold Enrichment

GO:0019886~ antigen processing and presentation of exogenous peptide antigen via MHC class II	6	2.05E-08	H2-EB1, IFI30, H2-AB1, H2-DMA, CD74, H2-DMB2	65.29
GO:0002504~ antigen processing and presentation of peptide or polysaccharide antigen via MHC class II	3	0.001468	H2-EB1, IFI30, H2-AB1, H2-DMA, CD74, H2-DMB2	65.29
GO:0051085~ chaperone mediated protein folding requiring cofactor	3	0.003127	MARCO, CFP, C1QB, LYN, H2-EB1, IFI30, H2-AB1, INPP5D, H2-DMA, C1QC, CD74, TLR8, LGALS9, CSF1R	5.60
GO:0051279~ regulation of release of sequestered calcium ion into cytosol	3	0.003633	H2-EB1, H2-AB1, CTSS, H2-DMA, CD74, H2-DMB2	16.93
GO:0045579~ positive regulation of B cell differentiation	3	0.00536	MMP9, IFI30, C1QL4, FTH1, LTA	23.80
GO:0030574~ collagen catabolic process	4	4.92E-04	PTPN6, LYN, LCK, NCKAP1L, IGKC	13.85
GO:0048147~ negative regulation of fibroblast proliferation	5	5.33E-05	CTSK, MMP9, CTSS, MMP14	25.39
GO:0035987~ endodermal cell differentiation	4	7.82E-04	ITGB2L, MMP9, MMP14, FN1	21.76
GO:0033189~ response to vitamin A	3	0.008913	HMOX1, PECAM1, TNFSF12, MMP14, MCAM, GJA5, FN1, ANXA2	5.06
GO:0045089~ positive regulation of innate immune response	3	0.008913	H2-EB1, H2-AB1, H2-DMA	50.78

GO:0016064~ immunoglobulin mediated immune response	3	0.009721	ITGB2L, LYN, CCR1, PPARG, LTB4R2, TNIP2, TLR8, LTA, CSF1R	3.93
GO:0019882~ antigen processing and presentation	6	2.63E-05	CORO1A, LYN, CCR1, MMP14, MCAM, CSF1R, FN1	5.15
GO:0050853~ B cell receptor signaling pathway	5	4.50E-04	H2-DMA, CD74, H2-DMB2	35.15
GO:0006909~ phagocytosis	4	0.004711	HHEX, PTPN6, PIM1, TACC3, CD74, LTA, CSF1R	4.74
GO:0051603~ proteolysis involved in cellular protein catabolic process	4	0.006395	PTPN6, CORO1A, LYN	32.64
GO:0006898~ receptor-mediated endocytosis	4	0.007026	CORO1A, PECAM1, IRF8, ANXA3	11.72
GO:0018108~ peptidyl-tyrosine phosphorylation	4	0.00876	MARCO, CFP, C1QB, LYN, LCK, IGKC, C1QC, TLR8, CSF1R	3.41
GO:0009612~ response to mechanical stimulus	4	0.009518	NCKAP1L, INPP5D, MMP14	26.88
GO:0006955~ immune response	16	2.55E-09	SCPEP1, CTSK, CTSC, CTSS	10.51
GO:0006935~ chemotaxis	5	0.007408	MARCO, MRC1, TFRC, FTH1	10.16
GO:0002376~ immune system process	14	1.21E-06	LYN, CCR1, NCKAP1L, LECT2, LGALS9	6.45
GO:0030335~ positive regulation of cell migration	7	0.002354	PTPN6, LYN, LCK, CSF1R	9.37
GO:0001525~ angiogenesis	8	0.001015	TYMS, PPARG, DNMT1	20.77
GO:0008283~ cell proliferation	7	0.003555	TLR8, CD74, LGALS9	20.77
GO:0006954~ inflammatory response	9	0.002032	LCK, PPARG, MMP14, SMPD2	9.09
GO:0045087~ innate immune response	9	0.004796	INPP5D, H2-DMA, CD74	19.87

Commonly regulated GO terms 7dpi (Figure 2B)

GO Term	Number of genes	p-value	Genes	Fold Enrichment
GO:2000323~ negative regulation of glucocorticoid receptor signaling pathway	4	1.27E-05	CRY2, PER1, ARNTL, CRY1	76.01
GO:0061469~ regulation of type B pancreatic cell proliferation	4	2.22E-05	NR1D1, NR4A1, NR4A3, ERRFI1	65.15
GO:0042754~ negative regulation of circadian rhythm	3	0.002042	CIPC, CRY2, CRY1	42.75
GO:0000188~ inactivation of MAPK activity	4	2.74E-04	DUSP4, DUSP1, DUSP16, DUSP8	30.40
GO:0001706~ endoderm formation	4	3.35E-04	DUSP5, DUSP4, DUSP2, DUSP1	28.50
GO:0035970~ peptidyl-threonine dephosphorylation	3	0.004704	DUSP5, DUSP4, DUSP1	28.50
GO:0043153~ entrainment of circadian clock by photoperiod	5	2.96E-05	RBM4B, CRY2, PER1, CRY1, SIK1	27.15
GO:0009416~ response to light stimulus	6	4.73E-06	FOS, FECH, DUSP1, JUND, PER1, JUNB	23.59
GO:0016126~ sterol biosynthetic process	4	0.001458	EBP, MSMO1, HMGCR, NSDHL	17.54
GO:0050772~ positive regulation of axonogenesis	4	0.001458	METRN, NIN, PLXNB1, PLXNB2	17.54
GO:0032922~ circadian regulation of gene expression	9	6.32E-08	RBM4B, RAI1, CRY2, NR1D1, CIART, PER1, ARNTL, BHLHE41, CRY1	16.55

GO:0032870~ cellular response to hormone stimulus	7	5.19E-06	FOS, DUSP1, JUN, JUND, FOSB, SIK1, JUNB	15.65
GO:0035914~ skeletal muscle cell differentiation	7	9.03E-06	EGR1, FOS, EGR2, ATF3, BTG2, DMRTA2, NR4A1	14.25
GO:0032570~ response to progesterone	5	3.98E-04	FOS, CLDN4, SOCS3, FOSB, JUNB	14.25
GO:0006695~ cholesterol biosynthetic process	4	0.002677	EBP, HMGCR, IDI2, NSDHL	14.25
GO:0071310~ cellular response to organic substance	4	0.002926	EGR1, EGR2, BCL2, NR4A1	13.82
GO:0051591~ response to cAMP	7	1.23E-05	FOS, DUSP1, JUN, JUND, PER1, FOSB, JUNB	13.53
GO:0009314~ response to radiation	4	0.003465	PLK3, JUN, JUND, JUNB	13.03
GO:0071549~ cellular response to dexamethasone stimulus	4	0.004062	FECH, EIF4E, H2-AB1, ERRFI1	12.33
GO:0007623~ circadian rhythm	11	5.47E-08	RBM4B, CRY2, NR1D1, CLDN4, DBP, JUN, JUND, PER1, ARNTL, BHLHE41, CRY1	11.10
GO:0042752~ regulation of circadian rhythm	5	0.001086	CRY2, NR1D1, NR1D2, PER1, CRY1	10.96
GO:0071277~ cellular response to calcium ion	5	0.001167	FOS, JUN, JUND, FOSB, JUNB	10.76
GO:0048511~ rhythmic process	12	1.57E-08	CIPC, RAI1, CRY2, NR1D1, NR1D2, DBP, CIART, PER1, ARNTL, BHLHE41, CRY1, SIK1	10.69
GO:0009612~ response to mechanical stimulus	6	2.95E-04	MEIS2, BTG2, JUN, JUND, FOSB, JUNB	10.21
GO:0045597~ positive regulation of cell differentiation	4	0.007496	SOCS3, JUN, PPP1R13L, JUNB	9.91
GO:0032868~ response to insulin	6	4.13E-04	EGR1, RBP4, EGR2, CRY2, SOCS3, CRY1	9.50

GO:0006694~ steroid biosynthetic process	5	0.002093	EBP, MSMO1, HMGCR, PBX1, NSDHL	9.19
GO:0051726~ regulation of cell cycle	9	7.02E-06	JUN, BCL2, JUND, RGCC, NPM1, ARNTL, CCNG1, GADD45B, JUNB	9.00
GO:0048661~ positive regulation of smooth muscle cell proliferation	5	0.005258	EGR1, HES5, HMGCR, JUN, NR4A3	7.13
GO:0034097~ response to cytokine	5	0.006246	FOS, JUN, BCL2, JUND, JUNB	6.79
GO:0007565~ female pregnancy	5	0.007644	FOS, CLDN4, FOSB, JUNB, LGALS9	6.41
GO:0016311~ dephosphorylation	6	0.002559	DUSP5, DUSP4, DUSP2, DUSP1, DUSP16, DUSP8	6.33
GO:0051384~ response to glucocorticoid	5	0.008575	KRAS, PTGDS, DUSP1, SOCS3, BCL2	6.20
GO:0006366~ transcription from RNA polymerase II promoter	7	0.001485	EGR1, FOS, EGR2, JUN, JUND, FOSB, JUNB	5.66
GO:0030182 neuron differentiation	6	0.005827	IER2, HES5, BTG2, EMX2, GFRA1, SMARCA1	5.22
GO:0006470~ protein dephosphorylation	6	0.007232	DUSP4, DUSP2, DUSP1, PPM1K, BCL2, DUSP8	4.96
GO:0043524~ negative regulation of neuron apoptotic process	7	0.003077	FAM134B, KRAS, BTG2, JUN, BCL2, NPM1, NR4A3	4.90
GO:0032496~ response to lipopolysaccharide	7	0.009811	FOS, SOCS3, JUN, JUND, GNG12, JUNB, LGALS9	3.84
GO:0000122~ negative regulation of transcription from RNA polymerase II promoter	20	2.38E-05	EGR1, HIST1H1C, EZH1, NR4A3, PPP1R13L, JUNB, PLK3, ATF3, CRY2, NR1D1, BTG2, HES5, JUN, JUND, PER1, BHLHE41, SCRT1, SIK1, CRY1, DNAJB5	3.09
GO:0042493~ response to drug	10	0.005132	FOS, FECH, SOCS3, JUN, BCL2, JUND, EMX2, FOSB, AK4, JUNB	3.09

GO:0006357~ regulation of transcription from RNA polymerase II promoter	10	0.008526	FOS, RAI1, ZFP395, ATF3, HIST1H1C, DBP, JUND, LDB2, FOSB, JUNB	2.85
GO:0043066~ negative regulation of apoptotic process	14	0.00146	EGR3, HMGCR, SOCS3, BNIP3, NR4A3, CCNG1, PDCD4, PLK3, PLK2, BTG2, DUSP1, JUN, BCL2, NPM1	2.81
GO:0045893~ positive regulation of transcription, DNA-templated	14	0.001756	EGR1, FOS, RAI1, EGR2, NR1D1, HES5, NR1D2, JUN, NPM1, NR4A1, NR4A3, NPAS4, ARNTL, SMARCA1	2.75
GO:0045892~ negative regulation of transcription, DNA-templated	14	0.001893	DUSP5, CIPC, CRY2, ATF3, NR1D1, HES5, NR1D2, JUN, CIART, PER1, ARNTL, BHLHE41, CRY1, PDCD4	2.72
GO:0045944~ positive regulation of transcription from RNA polymerase II promoter	23	5.81E-05	EGR1, RAI1, EGR2, CCPG1, EGR4, EZH1, NR4A1, LDB2, FOSB, ARNTL, NPAS4, NR4A3, JUNB, FOS, ATF3, MEIS2, HES5, DBP, JUN, JUND, RGCC, PER1, PBX1	2.62

Exclusively regulated GO terms in nostril 1dpi (Figure 2B)

GO Term	Number of genes	p-value	Genes	Fold Enrichment
GO:0031953~ negative regulation of protein autophosphorylation	4	1.65E-04	JUN, NLRP12, ERRFI1, MVP	35.25

GO:0030195~ negative regulation of blood coagulation	3	0.00758 2	THBD, APOH, ANXA5	22.37
GO:0016338~ calcium-independent cell-cell adhesion via plasma membrane cell-adhesion molecules	4	0.00183 5	CLDN7, CLDN4, ESAM, CLDN11	16.15
GO:0008652~ cellular amino acid biosynthetic process	4	0.00207	ADI1, MTHFD1, CTH, ASS1	15.51
GO:0032570~ response to progesterone	4	0.00794 7	CLDN4, C3, FOSL1, TGFB1	9.69
GO:0042130~ negative regulation of T cell proliferation	4	0.00794 7	MAD1L1, CEBPB, SDC4, TGFB1	9.69
GO:0007596~ blood coagulation	6	0.00172 8	THBD, FGA, C3, APOH, TFPI, ANXA5	6.92
GO:0042127~ regulation of cell proliferation	8	0.00909	SAT1, TNFRSF9, PLA2G4A, SGK1, PRG4, EGLN3, NDRG1, TGFB1	3.41
GO:0055114~ oxidation-reduction process	17	0.00168 6	EGLN3, DHRSX, PRDX1, PIPOX, MTHFD1L, GLDC, FMO4, MTHFD1, ADI1, RDH10, CYP27B1, DIO2, MARC2, CH25H, HSD17B3, LOXL1, BCO2	2.43

Exclusively regulated GO terms in nostril 2dpi (Figure 2B)

GO Term	Number of genes	p-value	Genes	Fold Enrichment
GO:0030048~ actin filament-based movement	3	0.00676	ACTC1, WASF2, MYH6	23.87
GO:0048873~ homeostasis of number of cells within a tissue	4	0.0025	GATA1, P2RX7, TEX15, KRAS	14.62
GO:0050766~positi ve regulation of phagocytosis	4	0.00524 8	MBL2, CYBA, PTK2, HSPA8	11.27
GO:0000187~ activation of MAPK activity	5	0.00225 1	P2RX7, ARRB1, EFNA1, TGFB3, PDE6G	9.01
GO:0007565~ female pregnancy	5	0.00417 9	OXT, TGFB3, CRH, HSD11B2, FOSL1	7.60
GO:0030324~ lung development	6	0.00245 3	CEBPA, RBP4, ARG1, GPC3, CRH, CP	6.39

GO:0010628~ positive regulation of gene expression	11	7.56E-04	RBM4B, ACTC1, WNT16, P2RX7, KRAS, TGFB3, CRH, IL2RG, ETV4, HSPA8, STAT3	3.71
GO:0042493~ response to drug	10	0.001618	ARG1, CYBA, ACTC1, P2RX7, NPPC, CRH, HSD11B2, FOSL1, DDIT3, STAT3	3.66

Exclusively regulated GO terms in nostril 3dpi (Figure 2B)

GO Term	Number of genes	p-value	Genes	Fold Enrichment
GO:0030199~ collagen fibril organization	3	0.008108	P4HA1, FOXC1, SERPINH1	21.78
GO:0042127~ regulation of cell proliferation	5	0.008011	STAT6, GM4907, CD81, EGLN3, NDRG1	6.23
GO:0007275~ multicellular organism development	11	0.002847	MSX3, DKK3, APLNR, BMP1, POGK, STK36, ACKR3, TMEM100, DNASE2A, PAQR5, FZD6	3.010

Exclusively regulated GO terms in nostril 7dpi (Figure 2B)

GO Term	Number of genes	p-value	Genes	Fold Enrichment
GO:1900153~ positive regulation of nuclear-transcribed mRNA catabolic process, deadenylation-dependent decay	3	0.006066	ZFP36L1, CNOT7, TOB1	25.17
GO:0055085~ transmembrane transport	9	0.008209	SLC25A4, SLC16A6, KCNA2, SLC22A7, SLC2A1, SLC41A1, ABCC1, SLC13A4, SLC26A2	3.12

Exclusively regulated GO terms in Skull 1dpi (Figure 2B)

GO Term	Number of genes	p-value	Genes	Fold Enrichment
GO:0018298~ protein-chromophore linkage	3	0.004291	CRY2, CRY1, RHO	29.78
GO:0045744~ negative regulation of G-protein coupled receptor protein signaling pathway	3	0.006013	RGS2, PLEK, CRY1	25.20

GO:0030218~ erythrocyte differentiation	6	7.34E-05	GATA1, ALAS2, LYN, HCLS1, KLF1, DNASE2A	13.65
GO:0030593~ neutrophil chemotaxis	6	5.03E-04	VAV3, PREX1, NCKAP1L, CSF3R, FCER1G, VAV1	9.10
GO:0009612~ response to mechanical stimulus	5	0.003249	MEIS2, PPARG, STAT1, EIF2AK2, JUNB	8.14
GO:0071407~ cellular response to organic cyclic compound	5	0.005106	PAK2, HSF1, CYP1A1, PPARG, STAT1	7.18
GO:0009615~ response to virus	5	0.007567	CYP1A1, PIM2, EIF2AK2, MX1, TLR8	6.42
GO:0007565~ female pregnancy	5	0.008874	SLC38A3, A2M, RXRA, CR1L, JUNB	6.13
GO:0007623~ circadian rhythm	6	0.003746	CRY2, MAT2A, RORB, RPE65, PER3, CRY1	5.79
GO:0048511~ rhythmic process	6	0.006335	CRY2, PPARG, CIART, RORB, PER3, CRY1	5.11

GO:0001701~ in utero embryonic development	12	9.22E-05	GATA1, ASCL2, HHEX, ACVRL1, HSF1, RXRA, TIE1, MYH6, KLF1, UBE2B, CR1L, JUNB	4.39
GO:0008285~ negative regulation of cell proliferation	13	2.33E-04	ACVRL1, LYN, TRPV2, RXRA, PPARG, PIM2, SKAP2, FRZB, GATA1, GPC3, HSF1, SCIN, EIF2AK2	3.65
GO:0002376~ immune system process	12	7.51E-04	PIK3CG, C1QA, LYN, MAP3K8, H2-AB1, EIF2AK2, TAPBPL, MX1, CR1L, TLR8, BTK, PSMB9	3.43
GO:0006915~ apoptotic process	13	0.006112	1810011O10RIK, TRPV2, PIM3, GZMB, PIM2, BCL2L13, STAT1, DNASE2A, BTK, PAK2, GADD45G, MAP3K8, GAPDH	2.48
GO:0000122~ negative regulation of transcription from RNA polymerase II promoter	16	0.003082	KLF5, BACH1, HCLS1, RXRA, PPARG, STAT1, JUNB, ASCL2, SUZ12, GATA1, HHEX, IFI27, CRY2, HSF1, PER3, CRY1	2.36

Exclusively regulated GO terms in Skull 2dpi (Figure 2B)

GO Term	Number of genes	p-value	Genes	Fold Enrichment
GO:0030335~ positive regulation of cell migration	5	0.009733	COL18A1, TNFAIP6, SYNE2, MYADM, PIK3R1	5.91

Exclusively regulated GO terms in Skull 3dpi (Figure 2B)

GO Term	Number of genes	p-value	Genes	Fold Enrichment
GO:0042512~ negative regulation of tyrosine phosphorylation of Stat1 protein	3	0.003553	SOCS3, SOCS1, IRF1	30.21
GO:0019885~ antigen processing and presentation of endogenous peptide antigen via MHC class I	3	0.005825	TAP2, ERAP1, TAPBP	24.17
GO:0042518~ negative regulation of tyrosine phosphorylation of Stat3 protein	3	0.008595	SUZ12, SOCS3, SOCS1	20.14
GO:0016126~ sterol biosynthetic process	9	1.41E-07	EBP, SC5D, MSMO1, HMGCR, CH25H, INSIG1, HMGCS1, NSDHL, FDFT1	13.94
GO:0045088~ regulation of innate immune response	4	0.002811	IRF7, IRF1, MYO1F, DHX58	13.43
GO:0006695~ cholesterol biosynthetic process	10	5.48E-08	EBP, APOA1, HMGCR, INSIG1, HMGCS1, LSS, IDI2, HSD17B7, NSDHL, FDFT1	12.59

GO:0008299~ isoprenoid biosynthetic process	4	0.00792 5	HMGCR, HMGCS1, IDI2, FDFT1	9.48
GO:0006694~ steroid biosynthetic process	13	3.24E-08	EBP, SC5D, MSMO1, HMGCR, HSD17B12, HMGCS1, LSS, FDFT1, CH25H, PBX1, SRD5A1, HSD17B7, NSDHL	8.45
GO:0071345~ cellular response to cytokine stimulus	6	0.00140 2	CXCR4, HCLS1, SOCS1, NLRP12, NFKBIA, STAT1	7.11
GO:0043124~ negative regulation of I-kappaB kinase/NF-kappaB signaling	7	4.20E-04	OLFM4, NLRC3, NLRP12, PYCARD, OPTN, STAT1, TANK	7.05
GO:0008202~ steroid metabolic process	13	1.04E-06	EBP, SC5D, MSMO1, APOA1, HMGCR, CH25H, APOF, SLC37A4, INSIG1, HMGCS1, SRD5A1, NSDHL, FDFT1	6.23
GO:0002479~ antigen processing and presentation of exogenous peptide antigen via MHC class I, TAP-dependent	5	0.00860 8	PSMB7, PSMA6, PSMB8, TAPBP, PSMB9	6.10
GO:0009617~ response to bacterium	5	0.00956 9	BAIAP2L1, NCF1, PYCARD, STAT1, FUCA2	5.92

GO:0006633~ fatty acid biosynthetic process	9	3.61E-04	ELOVL1, SC5D, MSMO1, PTGDS, CH25H, HSD17B12, ELOVL2, FADS3, SCD4	5.11
GO:0008203~ cholesterol metabolic process	10	3.57E-04	EBP, APOA1, HMGCR, APOF, CH25H, INSIG1, HMGCS1, ABCA2, NSDHL, FDFT1	4.53
GO:0050728~ negative regulation of inflammatory response	9	0.001516	ZFP36, ABR, APOA1, SHARPIN, SOCS3, NLRP12, ACP5, NLRP3, ADA	4.12
GO:0031100~ organ regeneration	7	0.007186	PKM, NRF1, APOA1, SOCS3, EIF4A1, SOCS1, CCNA2	4.09
GO:0007565~ female pregnancy	9	0.00163	FOS, SLC38A3, OXT, CRH, HSD11B2, IGF2, IGFBP2, JUNB, EPN1	4.07
GO:0032868~ response to insulin	7	0.008806	RBP4, SLC27A1, GRB10, SOCS3, HSD11B2, OGT, PCK1	3.92
GO:0009615~ response to virus	8	0.005122	HYAL2, TLR13, RSAD2, EIF2AK2, MX1, MX2, DHX58, CXCL10	3.79
GO:0051384~ response to glucocorticoid	8	0.007852	PTGDS, SOCS3, OXT, MSTN, HSD11B2, FAS, IGFBP2, CDO1	3.50

GO:0051607~ defense response to virus	14	2.90E-04	PTPRC, HYAL2, RSAD2, TRIM25, IFIT3B, NLRP3, CXCL10, ISG15, PYCARD, IRF1, EIF2AK2, MX1, MX2, DHX58	3.36
GO:0016311~ dephosphorylation	9	0.005419	DUSP5, PNKP, MTMR3, PTPRC, DUSP3, PTPRG, DUSP16, DUSP22, ACP5	3.36
GO:0032496~ response to lipopolysaccharide	17	6.46E-05	ABR, SOCS3, SOCS1, NFKBIA, ACP5, STAT1, CXCL11, JUNB, TRIB1, CXCL10, TNFRSF9, FOS, NRF1, NLRC3, CXCL13, FAS, EIF2AK2	3.29
GO:0002376~ immune system process	27	3.40E-06	MBL2, RSAD2, IL34, LGR4, RNF125, CASP4, FGA, TAP2, PYCARD, ERAP1, MR1, MX1, MX2, DHX58, HERC6, TLR13, TRIM25, NLRP3, PSMB8, PSMB9, C1QA, SARM1, IRF7, IRF1, TAPBPL, EIF2AK2, SEMA4A	2.85
GO:0006629~ lipid metabolic process	30	4.47E-06	ENPP6, SLC27A1, SC5D, HMGCR, HSD17B12, HMGCS1, ST8SIA1, LSS, ASAH1, FDFT1, ELOVL1, MTMR3, APOA1, CH25H, APOF, ELOVL2, PLCH1, INSIG1, SRD5A1, SCD4, HSD17B7, NSDHL, EBP, MSMO1, PLD4, FADS3, CRAT, PCK1, PTGDS, LIPG	2.63
GO:0045087~ innate immune response	22	0.001131	MBL2, TLR13, HERC6, RSAD2, TRIM25, NLRP3, IL34, LGR4, C1QA, CYBB, SARM1, CASP4, FGA, IRF7, PYCARD, IRF1, FCER1G, MR1, EIF2AK2, MX1, MX2, DHX58	2.20

GO:0042493~ response to drug	19	0.00510 8	MAT2A, SOCS3, LGALS1, SOCS1, GRIN2A, HMGCS1, IGF2, STAT1, JUNB, ADA, FOS, CYBB, MTHFR, APOA1, CRH, HSD11B2, SRD5A1, FAS, IGFBP2	2.07

Exclusively regulated GO terms in Skull 7dpi (Figure 2B)

GO Term	Number of genes	p-value	Genes	Fold Enrichment
GO:0042493~ response to drug	10	3.15E-04	TXNIP, COL18A1, ARG1, MMP9, NPPC, CRH, HMGCS1, GNPAT, SRD5A1, ABCC2	4.59
GO:0014074~ response to purine-containing compound	3	6.98E-04	HIF1A, SLC6A1, HMGCS1	72.61
GO:0000122~ negative regulation of transcription from RNA polymerase II promoter	13	0.00125 5	ZFP36, TXNIP, STAT6, KLF5, IFI27, HIF1A, BCL11A, MNT, BCL6B, BHLHE40, NFIL3, TAGLN3, FOXP2	2.98
GO:0048546~ digestive tract morphogenesis	3	0.00487 5	EGFR, HIF1A, STRA6	28.24
GO:0006694~ steroid biosynthetic process	4	0.00572 3	HSD3B4, HMGCS1, LSS, SRD5A1	10.93

GO:0071353~ cellular response to interleukin-4	3	0.00930 8	ARG1, NFIL3, TUBA1B	20.33
GO:0043627~ response to estrogen	4	0.00967 5	ADRB2, CRH, MSTN, ABCC2	9.04

Table S5. RNAseq data depicting the Gene Ontology terms Biological Processes significantly enriched in an ECM-related gene set (69 genes) regulated exclusively 3 days after skull injury (Related to Figure 2E).

Pathway	Number of genes	p-value	Fold Enrichment
immune system process	21	3.65E-12	5.96
immune response	14	9.70E-09	6.3
immune effector process	7	3.20E-07	12.08
positive regulation of immune system process	10	5.80E-07	6.9
regulation of immune system process	12	3.34E-06	4.78
protein activation cascade	6	4.13E-06	11.29
positive regulation of immune response	6	2.38E-05	8.87
complement activation	4	7.12E-05	13.8
T cell activation	4	7.12E-05	13.8
regulation of immune response	6	2.43E-04	6.21
humoral immune response	4	3.08E-04	10.35
proteolysis	17	4.09E-04	2.41
response to stimulus	24	5.34E-04	1.97
activation of immune response	4	8.58E-04	8.28

Table S6. Microarray data illustrating the GO Terms Biological Processes enriched in genes exclusively regulated 3 days after skull injury (related to Figure 2H).

GO Term	Number of genes	p-value	Genes	Fold Enrichment
GO:0051607~ defense response to virus	5	7.77E-04	PTPRC, CARD9, RSAD2, NLRP3, TLR8	11.31
GO:0042439~ ethanolamine and derivative metabolic process	5	0.001623 474	ENPP6, PCYT1A, ETNK2, FABP5, PNPLA6	9.43
GO:0045667~ regulation of osteoblast differentiation	5	0.006589 917	TWSG1, UCMA, SOX2, CD276, CTNNB1	6.53
GO:0009615~ response to virus	10	3.75E-04	PTPRC, CARD9, ISG15, IRF7, RSAD2, MX1, NLRP3, EIF2AK2, MX2, TLR8	4.47
GO:0006576~ biogenic amine metabolic process	9	0.002380 975	SAT1, ENPP6, SAT2, GRIN2A, SLC22A4, PCYT1A, ETNK2, FABP5, PNPLA6	3.82
GO:0045087~ innate immune response	10	0.004257 998	C1QA, CIITA, MBL2, PTPN6, C4B, TLR13, MX1, MX2, TLR8, DHX58	3.17
GO:0006006~ glucose metabolic process	13	8.73E-04	RBP4, PDK2, SLC37A4, PHKA1, PPP3R2, IGF2, CAR5A, CPT1A, PCK1, PPP1R3E, PPP1R3C, FABP5, MDH2	3.15
GO:0002252~ immune effector process	11	0.004026 952	C1QA, MBL2, PTPN6, PTPRC, CARD9, C4B, IRF7, RSAD2, NLRP3, H2-DMA, TLR8	2.96
GO:0006575~ cellular amino acid derivative metabolic process	12	0.002987 015	SAT1, ENPP6, PCYOX1L, P4HA1, SAT2, GRIN2A, SLC22A4, PCYT1A, ETNK2, CDO1, FABP5, PNPLA6	2.89

GO:0019318~ hexose metabolic process	13	0.004260 456	RBP4, PDK2, SLC37A4, PHKA1, PPP3R2, IGF2, CAR5A, CPT1A, PCK1, PPP1R3E, PPP1R3C, FABP5, MDH2	2.61
GO:0007507~ heart development	17	9.31E-04	NOX4, NRP2, RBP4, GJA1, GJA5, SUFU, CTNNB1, GJC1, ALDH1A2, SALL4, OSR1, JMJD6, PBRM1, RARA, FOXC1, NCOR2, ACVR1	2.59
GO:0001701~ in utero embryonic development	20	3.55E-04	GNA13, FGFR1, CEBPB, ESRRB, SOCS3, MYO1E, GJA1, MBNL1, CAPN2, UBE2B, ASCL2, SIN3A, SALL4, PDGFRA, BTF3, ETNK2, HS6ST1, FOXC1, NCOR2, ACVR1	2.54
GO:0009611~ response to wounding	25	9.59E-05	GNA13, CIITA, PTPN6, MBL2, F10, C4B, F13A1, TLR13, GRIN2A, GJA1, ABHD2, NLRP3, CXCL11, TLR8, C1QA, FGA, SLC7A2, HRH4, CD81, PYCARD, APOH, NEFL, KDM6B, ACVR1, FN1	2.44
GO:0001775~ cell activation	17	0.002560 972	FYB, GNA13, PTPRC, CRIP3, LRRC8A, SKAP2, CTNNB1, WBP2NL, NLRC3, FGA, JMJD6, SLC7A2, BCL11A, IRF1, NDRG1, H2-DMA, LCP2	2.35
GO:0032787~ monocarboxylic acid metabolic process	18	0.002481 588	SLC27A1, RBP4, SLC37A4, ABHD5, PRKAG2, ACACA, CAR5A, ACOT5, MTHFD1L, CPT1A, PCK1, ALDH1A2, PTGDS, P4HA1, CH25H, ELOVL2, SLC22A4, GPAM	2.28

GO:0001568~ blood vessel development	16	0.005784 728	GNA13, FGFR1, SOCS3, MYO1E, GJA1, GJA5, MMP2, PNPLA6, GJC1, CTNNB1, ALDH1A2, JMJD6, HS6ST1, FOXC1, PPAP2B, ACVR1	2.23
GO:0035295~ tube development	17	0.005065 508	GNA13, FGFR1, RBP4, GJA1, GJA5, LGR4, SUFU, CTNNB1, ALDH1A2, JMJD6, PDGFRA, RARA, HS6ST1, FOXC1, ADAMTS2, FOXD1, ACVR1	2.19
GO:0043009~ chordate embryonic development	27	3.03E-04	GNA13, FGFR1, RBP4, GJA1, SUFU, SIN3A, BTF3, HS6ST1, ETL4, ETNK2, ALX3, CEBPB, SOCS3, ESRRB, MYO1E, GAS1, MBNL1, CAPN2, UBE2B, ASCL2, ACVR2A, SALL4, PDGFRA, FOXC1, RIPPLY2, NCOR2, ACVR1	2.18
GO:0001944~ vasculature development	16	0.007190 723	GNA13, FGFR1, SOCS3, MYO1E, GJA1, GJA5, MMP2, PNPLA6, GJC1, CTNNB1, ALDH1A2, JMJD6, HS6ST1, FOXC1, PPAP2B, ACVR1	2.17
GO:0009792~ embryonic development ending in birth or egg hatching	27	3.48E-04	GNA13, FGFR1, RBP4, GJA1, SUFU, SIN3A, BTF3, HS6ST1, ETL4, ETNK2, ALX3, CEBPB, SOCS3, ESRRB, MYO1E, GAS1, MBNL1, CAPN2, UBE2B, ASCL2, ACVR2A, SALL4, PDGFRA, FOXC1, RIPPLY2, NCOR2, ACVR1	2.16
GO:0006066~ alcohol metabolic process	22	0.002280 423	PDK2, RBP4, ENPP6, PHKA1, SLC37A4, GRIN2A, PPP3R2, IGF2, CAR5A, GDPD4, PNPLA6, CPT1A, FDFT1, PCK1, PPP1R3E, PPP1R3C, CH25H, APOF, PCYT1A, ETNK2, FABP5, MDH2	2.07

Table S7. RNAseq data showing the GO Terms Biological Processes (PANTHER) upregulated after brain injury in zebrafish Olig2^{+} cells (Related to Figure S7C, GO Terms marked in red are shown in the graph S7C).

GO Term	Number of genes	FDR-value	Fold Enrichment
toll-like receptor signaling pathway	12	4,59E-05	9,15
pattern recognition receptor signaling pathway	14	5,79E-06	9,08
activation of innate immune response	14	1,86E-05	7,89
response to chemokine	16	1,27E-04	5,32
innate immune response	27	6,74E-07	4,55
response to lipopolysaccharide	13	4,43E-03	4,44
immune response	80	1,37E-20	4,41
defense response	60	5,34E-13	3,83
response to cytokine	32	3,16E-06	3,55
cell migration	75	5,83E-07	2,25
carbohydrate metabolic process	36	9,54E-03	2,12
chemotaxis	48	7,46E-04	2,12
innate immune response-activating signal transduction (GO:0002758)	14	5,66E-06	9,08
positive regulation of defense response (GO:0031349)	17	4,24E-06	6,89
positive regulation of innate immune response (GO:0045089)	16	1,37E-05	6,69
positive regulation of response to biotic stimulus (GO:0002833)	16	1,34E-05	6,69
immune response-activating signal transduction (GO:0002757)	27	2,38E-09	6,48
activation of immune response (GO:0002253)	32	1,04E-10	6,19
immune response-regulating signaling pathway (GO:0002764)	27	6,94E-09	6,04
neutrophil migration (GO:1990266)	24	1,55E-07	5,76
neutrophil chemotaxis (GO:0030593)	22	7,03E-07	5,71
humoral immune response (GO:0006959)	13	6,81E-04	5,62

chemokine-mediated signaling pathway (GO:0070098)	16	7,51E-05	5,61
granulocyte migration (GO:0097530)	24	3,08E-07	5,46
myeloid leukocyte migration (GO:0097529)	26	8,72E-08	5,44
leukocyte chemotaxis (GO:0030595)	25	1,83E-07	5,4
granulocyte chemotaxis (GO:0071621)	22	1,56E-06	5,38
cellular response to chemokine (GO:1990869)	16	1,29E-04	5,32
immune response-activating cell surface receptor signaling pathway (GO:0002429)	13	1,14E-03	5,27
positive regulation of immune response (GO:0050778)	36	1,83E-10	5,19
positive regulation of response to external stimulus (GO:0032103)	18	6,47E-05	4,97
leukocyte migration (GO:0050900)	26	3,20E-07	4,96
positive regulation of multi-organism process (GO:0043902)	17	1,44E-04	4,9
response to virus (GO:0009615)	19	4,56E-05	4,83
cell chemotaxis (GO:0060326)	32	1,61E-08	4,72
immune response-regulating cell surface receptor signaling pathway (GO:0002768)	13	2,90E-03	4,68
inflammatory response (GO:0006954)	37	1,09E-09	4,66
regulation of innate immune response (GO:0045088)	16	5,05E-04	4,61
positive regulation of immune system process (GO:0002684)	44	2,76E-11	4,56
regulation of response to biotic stimulus (GO:0002831)	17	3,55E-04	4,5
regulation of immune response (GO:0050776)	39	2,48E-09	4,25
response to molecule of bacterial origin (GO:0002237)	13	6,38E-03	4,21
response to external biotic stimulus (GO:0043207)	66	8,31E-15	3,98
response to other organism (GO:0051707)	66	6,23E-15	3,98

cytokine-mediated signaling pathway (GO:0019221)	29	2,12E-06	3,96
response to bacterium (GO:0009617)	33	2,55E-07	3,96
response to biotic stimulus (GO:0009607)	66	7,38E-15	3,94
regulation of defense response (GO:0031347)	21	1,81E-04	3,89
defense response to other organism (GO:0098542)	41	6,19E-09	3,88
regulation of immune system process (GO:0002682)	63	9,12E-14	3,85
cellular response to cytokine stimulus (GO:0071345)	29	3,41E-06	3,84
immune effector process (GO:0002252)	17	2,19E-03	3,74
carbohydrate derivative catabolic process (GO:1901136)	17	3,51E-03	3,56
regulation of multi-organism process (GO:0043900)	18	2,30E-03	3,54
leukocyte activation (GO:0045321)	15	9,82E-03	3,47
immune system process (GO:0002376)	150	3,11E-25	3,02
multi-organism process (GO:0051704)	71	1,42E-10	2,89
positive regulation of hydrolase activity (GO:0051345)	28	9,61E-03	2,36
positive regulation of response to stimulus (GO:0048584)	77	1,11E-06	2,16
response to external stimulus (GO:0009605)	104	4,25E-09	2,16
localization of cell (GO:0051674)	75	3,20E-06	2,14
cell motility (GO:0048870)	75	3,12E-06	2,14
taxis (GO:0042330)	48	1,70E-03	2,04

Table S8. RNAseq data depicting the Gene Ontology terms Biological Processes (PANTHER) significantly enriched in a gene set (597 genes) with normalized expression after Tlr1/2&Cxcr3 inhibitor treatment in zebrafish. (Related to Figure 5B).

Pathway	Number of genes	p-value	Fold Enrichment
mitotic cell cycle	14	0,00812	2,18
myelination	5	0,00634	4,61
regulation of endopeptidase activity	7	0,00256	4,05
regulation of apoptotic process	17	0,00176	2,4
ensheathment of neurons	6	0,00152	5,28
response to chemokine	6	0,000869	5,96
response to tumor necrosis factor	6	0,000769	6,12
cellular response to interferon-gamma	5	0,0006	8,42
regulation of GTPase activity	12	0,000469	3,32
cytokine-mediated signaling pathway	10	0,000313	4,08
response to interleukin-1	6	0,000251	7,75
cell chemotaxis	10	0,000178	4,4
innate immune response	11	0,0000126	5,54
defense response	21	0,000000244	4,01
immune system process	43	5,37E-08	2,59

Table S9. RNAseq data depicting the Gene Ontology terms Biological Processes (PANTHER) significantly enriched in a gene set still regulated after inhibitor treatment. (Related to Figure 5).

Pathway	Number of genes	p-value	Fold Enrichment
immune system process	9	4.36E-05	6.70
immune response	41	1.7E-13	3.96
proteolysis involved in cellular protein catabolic process	19	4.64E-13	9.43
inflammatory response	28	5.06E-13	5.62
innate immune response	25	1.01E-09	4.56
regulation of cell proliferation	19	2.07E-07	4.48
carbohydrate metabolic process	22	9.59E-07	3.56
peptidyl-tyrosine autophosphorylation	10	6.05E-06	7.26
proteolysis	40	1.14E-05	2.14
response to lipopolysaccharide	10	5.44E-05	5.66
transmembrane receptor protein tyrosine kinase signaling pathway	14	5.98E-05	3.88
